# Recent Development of Carbon-Nanotube-Based Solar Heat Absorption Devices and Their Application

**DOI:** 10.3390/nano12213871

**Published:** 2022-11-02

**Authors:** Saiful Islam, Hiroshi Furuta

**Affiliations:** 1 School of Systems Engineering, Kochi University of Technology, Kochi 782-8502, Japan; 2 Center for Nanotechnology, Research Institute, Kochi University of Technology, Kochi 782-8502, Japan

**Keywords:** CNTs, solar heat absorption, energy storage, thermoelectric generator, steam generator, water heater

## Abstract

Population growth and the current global weather patterns have heightened the need to optimize solar energy harvesting. Solar-powered water filtration, electricity generation, and water heating have gradually multiplied as viable sources of fresh water and power generation, especially for isolated places without access to water and energy. The unique thermal and optical characteristics of carbon nanotubes (CNTs) enable their use as efficient solar absorbers with enhanced overall photothermal conversion efficiency under varying solar light intensities. Due to their exceptional optical absorption efficiency, low cost, environmental friendliness, and natural carbon availability, CNTs have attracted intense scientific interest in the production of solar thermal systems. In this review study, we evaluated CNT-based water purification, thermoelectric generation, and water heating systems under varying solar levels of illumination, ranging from domestic applications to industrial usage. The use of CNT composites or multilayered structures is also reviewed in relation to solar heat absorber applications. An aerogel containing CNTs was able to ameliorate water filtering performance at low solar intensities. CNTs with a Fresnel lens improved thermoelectric output power at high solar intensity. Solar water heating devices utilizing a nanofluid composed of CNTs proved to be the most effective. In this review, we also aimed to identify the most relevant challenges and promising opportunities in relation to CNT-based solar thermal devices.

## 1. Introduction

The shortage of fresh water, electricity, and hot water for residential and industrial uses has been one of the greatest threats to human progress [1,2]. Renewable energy sources include hydrogen, the sun, wind, and the Earth’s geothermal heat. Solar energy exhibits a wide range of benefits, the most notable of which are its abundance, availability, and dependability. Sunlight is capable of heating water, generating electricity, and desalinating seawater. Despite the multiple benefits of solar energy, thermal efficiency remains poor. Modifying the solar absorption material could enhance the performance of solar thermal devices [3]. Superior materials for solar absorption are characterized by hydrophilicity, porosity, solar-thermal conversion efficiency, and self-floating properties [4]. Almost all solar applications require absorbing materials to absorb solar energy and to carry out thermal management programs. It is always necessary to use absorbent materials to receive solar radiation and implement heat management programs. A solar thermal application also requires a light-absorbing and thermally converting transmitter, which is subsequently delivered to a liquid to enhance the temperature. CNTs have been proven to be effective solar absorbers in solar thermal systems due to their broad absorption spectrum [5].

Carbon nanotubes (CNTs) were first reported by Sumio Iijima in 1991 [6]. The unique optical [7], electrical [8], and mechanical [9] properties of CNTs have attracted researchers to expand their research and use them in renewable energy applications. CNTs are also known as the darkest materials on Earth as they are capable of absorbing most incident light [10]. In the realm of solar thermal technology, black body absorbers have demonstrated the potential to be used in actual applications.

Previous research has shown that CNTs have effective and opaque characteristics, absorb light from the visible to infrared wavelengths. In 2004, researchers investigated the optical effect of the unique π interband transition of the CNT structure. They found that multi-wall carbon nanotubes (MWCNTs) with a diameter of 60 nm exhibited a narrow optical band gap of 100 meV [11,12]. Wang et al. [13] also reported that CNTs at the height of 80 µm absorbed 99.2% of light with wavelengths ranging from 400 nm to 1800 nm. CNTs have also shown low optical reflectance in regard to the refractive index, resulting from their hollow porous structure [14]. Miyaji et al. [15] revealed that the use of a fishnet metamaterial composed of CNTs and carbon films with a height of 0.58 µm increased the absorption and reduced the reflectance and transmittance of infrared radiation (IR). Chu et al. [16] highlighted CNTs’ exceptional optical nonlinearity and wideband absorption characteristics.

Researchers have investigated the unique thermal properties which arise from the tubular shape of CNTs. Yang et al. [17] used the pulse photothermal reflectance technique to achieve 15 W/m·K thermal conductivity with 10–50 µm thick MWCNTs. Ivanov et al. [18] showed that a vertically aligned array of CNTs with a thickness of 2 nm displayed a very high anisotropic thermal diffusivity ratio. Furthermore, a one-dimensional heat diffusion model has been implemented, with researchers observing that the thermal conductivity of MWCNTs increased from 200 to 400 W/m·K above the ambient temperature [19]. Moreover, single-wall carbon nanotubes (SWCNTs) with a fixed diameter and length show excellent thermal conductivity at room temperature [20]. Fuji et al. [21] measured the thermal conductivity of SWCNTs using a T-nanosensor. They tested the different diameters of CNTs and T-shaped nanosensors and recorded a thermal conductivity of 2000 W/m·K for SWCNTs of 9.8 nm in diameter.

CNTs have been designated as candidate materials for thermo-optical applications due to their unique optical and thermal characteristics [22]. Advantages of chemical stability and higher efficiencies of thermal heat conversion of CNT-based materials are reported [23,24] in nanofluids material, compared to the emerging materials such as Al_2_O_3,_ Ag, SiO_2_, ZnO, TiO_2_ and graphene. On other hand, CNTs have been criticized as ineffective when not perfectly formed. For example, Cui et al. [25] explained that the vacancy defect of crystals decreases the thermal conductivity of CNTs. Caccamo et al. [26] experimentally revealed the thermal behavior of CNTs using Fourier transform infrared and Raman spectroscopy analysis. They found that the relaxation of temperature decreased the mechanical and thermal properties of CNTs. Density and alignment have a remarkable impact on the thermal behavior of CNTs. Highly aligned CNTs increase electrical and thermal conductivity [27]. Zhan et al. [28] fabricated a highly densified and aligned CNT film, followed by a pressing and stretching process, and achieved 700 W/m·K thermal conductivity. They explained the importance of the high alignment of CNTs in the film in order to achieve high thermal conductivity. Wang et al. [29] performed the noncontact thermal characterization of MWCNTs and observed low thermal conductivity due to the poor structural quality of CNTs. Furthermore, Zhang et al. [30] fabricated Bucky paper (prepared with the pressing of highly aligned CNT films) with a density of 1.39 mg/cm^3^, and the thermal conductivity reached 766 W/m·K. Yang et al. [31] performed 3D microscopic analysis of SWCNTs, MWCNTs, and an MWCNT sponge. They concluded that the density of CNTs has a significant effect on thermal conductivity.

Although most experiments have been conducted in environments with 1 kW/m^2^ of sunlight (1 sun), this illumination level is insufficient for practical applications because solar irradiance can change from one place to another depending on the weather and climate conditions. Actual applications of solar thermal devices operating in environments with low (<1 kW/m^2^) and high (>1 kW/m^2^) radiation have substantial effects [29,30,32,33], which are described in the following sections in detail. In this review we provide a deep insight into CNT-based water purification, thermoelectric generation, water heating systems, and their practical implementation under different levels of solar power. In this article, we also aimed to identify a possible method of overcoming gaps in the research by investigating the use of CNT materials for thermal energy applications, thus addressing both UN Sustainable Development Goal 6: Clean water and sanitation [34] and Goal 7: Affordable and clean energy [35].

## 2. CNT-Based Solar Heat Conversion Devices

**Figure 1:**
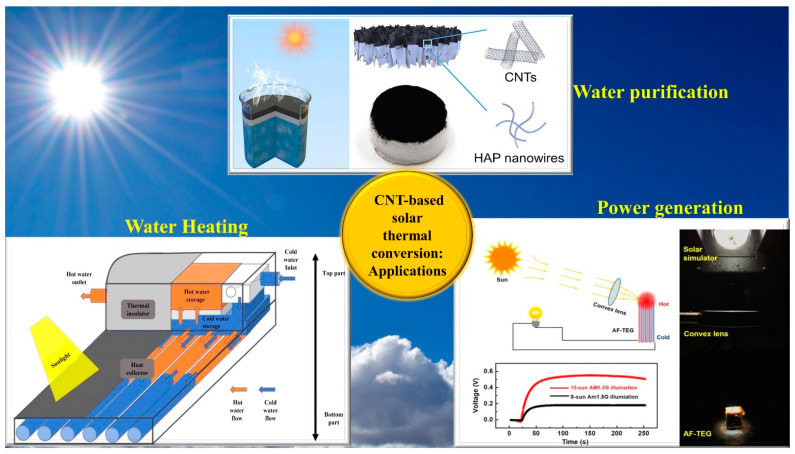
Recent applications of CNT-based solar thermal devices for water purification, power generation, and water heating (reproduced with permission, Copyright 2021 Elsevier [36]; reproduced with permission, Copyright 2019 Elsevier [37].)

The most important benefits derived from the thermal and optical properties of CNTs have been discussed in the preceding section. CNTs, a relatively new material with excellent optical and thermal properties, can be used in various solar technologies. In this section, we discuss the use of CNTs as solar absorbers due to their inherent high-bandwidth light absorption properties. As illustrated in Figure 1, CNT-based solar absorbers can provide high evaporation efficiency in solar water purification devices, improve power output in solar thermoelectric generators, and enhance thermal efficiency in solar water heating devices.

### 2.1. Water Purification

**Figure 2:**
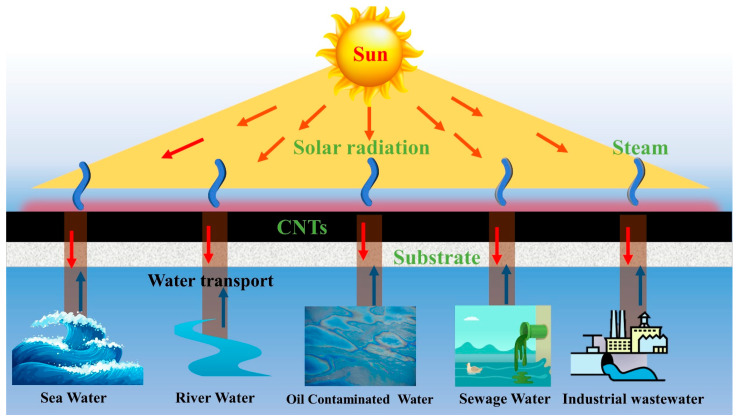
Schematic illustration of CNTs’ photothermal-conversion-based solar water purification process.

Materials composed of CNTs have several advantageous properties, including high light absorption, a large specific surface area, and excellent thermal conductivity. Due to these qualities, CNT materials are ideal for water filtration systems powered by the sun [38]. Figure 2 illustrates the process of solar-powered water purification, including light absorption, photothermal conversion, water transfer, and evaporation. This process is used to clean wastewater, contaminated water, and seawater. Bottom, bulk, and interfacial heat transfers are the three primary types of solar heating. Solar interfacial heating is the most effective method for water purification due to its high evaporation efficiency, low cost, and long lifespan [39]. In this section, we describe the comprehensive and in-depth studies conducted on the evaporation efficiency and applications of solar water purification (SWP) at high and low sun intensities.

Solar water purification methods have focused on increasing evaporation efficiency. The efficiency of evaporation can be quantified by dividing the amount of solar radiation by the amount of thermal energy stored in the generated vapor.

(1) $$\eta =\frac{mh_{LV}}{Q_{in}}$$

Here, η = evaporation efficiency, m = the mass flux, $h_{LV}$ = total vaporization enthalpy of the water (sensible heat + phase change enthalpy), and $Q_{in}$ = total input solar thermal energy.

**Figure 3:**
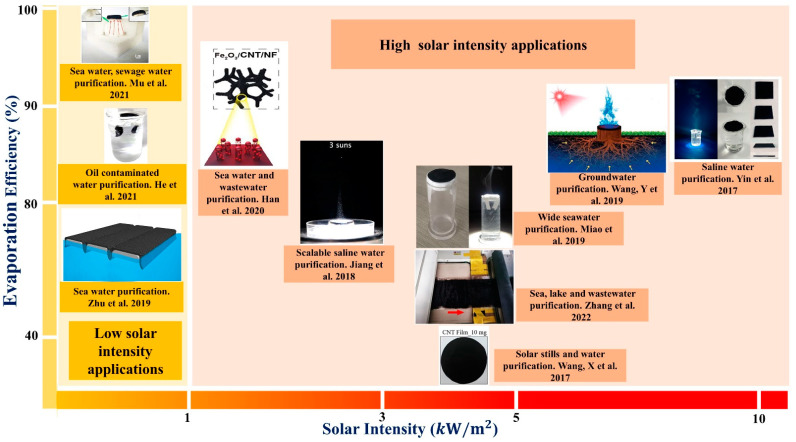
Mapping of applications for CNT-based solar thermal devices based on solar intensity in relation to evaporation efficiency (reproduced with permission, Copyright 2020 Elsevier [40]; reproduced with permission, Copyright 2017 The Royal Society of Chemistry [41]; reproduced with permission, Copyright 2019 American Chemical Society [42]; reproduced with permission, Copyright 2020 American Chemical Society [43]; reproduced with permission, Copyright 2018 American Chemical Society [44]; reproduced with permission, Copyright 2019 Elsevier [45]; reproduced with permission, Copyright 2022 American Chemical Society [46]; reproduced with permission, Copyright 2017 Elsevier [47]; reproduced with permission, Copyright 2018 John Wiley and Sons [48]; reproduced with permission, Copyright 2017 American Chemical Society [49]).

Several approaches have been presented to achieve a high evaporation efficiency under solar irradiation, whereas practical applications for water purification have received considerable attention, as shown in Figure 3. Most research has been conducted under low sunlight intensity. The low solar illumination is primarily constrained by the solar energy input, limiting evaporation efficiency. On the other hand, different materials (aerogels or membranes) used in combination with CNTs can improve evaporation efficiency at low solar intensity.

**Figure 4:**
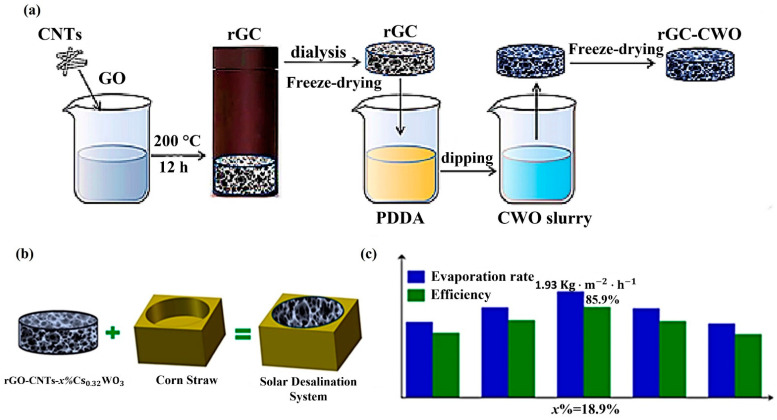
(**a**) Illustration of the preparation process of rGC-CWO aerogel. (**b**) Schematic diagram of rGC-CWO/CS evaporator. (**c**) The corresponding systems’ evaporation rates and efficiency (reproduced with permission, Copyright 2022 Elsevier [54]).

Aerogel is one possible solution to improve evaporation efficiency. Zhou et al. [50] reported that low vaporization enthalpy is an effective strategy for functioning solar water purification at low solar irradiation, which also follows Equation (1). Recently, Mu et al. [51] introduced nanoporous super-wetting hollow carbon nanotube (HCNT) aerogels that were super-hydrophilic following sulfuric acid treatment. This device exhibited an evaporation efficiency of 86.8% when exposed to one-sun illumination (1 sun = 1 kW/m^2^). Qin et al. [37] also employed a bilayer aerogel technique with hydrophilic ultralong hydroxyapatite (HAP) nanowire aerogel and a hydrophobic CNT coating to boost evaporation efficiency to 89.4% under one-sun illumination. They validated the possible applicability of the device in the removal of heavy metal ions from saltwater and wastewater. Li et al. [52] proposed a double-layer concave solar evaporator with an absorbing layer composed of Ti_2_O_3_ nanoparticles and CNT aerogel. They enhanced the effectiveness of evaporation to 92.4% and demonstrated the device’s capacity to cleanse water from wastewater containing chemicals or heavy ions. Xu et al. [53] prepared a modified shape, referred to as organohydrgel, doped with CNTs to purify oil-contaminated water under low sunlight illumination conditions. Li et al. [54] reported on the use of carbon-based hydrogels, rGC-constituted rGOs (reduced graphene oxides), and CNTs. They were synthesized using the hydrothermal technique, and cesium tungsten bronze nanoparticles were deposited on the surface of rGCs using an impregnation procedure. They used polydiallyl dimethyl ammonium chloride (PDDA) solution to alter the surface potential of the rGC aerogel. Nano-tungsten bronze aerogels (rGC-CWO) were prepared using the freeze-drying technique, as shown in Figure 4a. Then, the rGC-CWO aerogels were implanted in a corn straw (CS) substrate to construct rGC-CWO/CS evaporators, as shown in Figure 4b. Figure 4c shows that the rGC-CWO composite aerogels emitted considerable heat, and the water evaporation efficiency increased significantly to 85.9% in purifying seawater under weaker solar intensity (1 sun).

The use of CNT membrane-based solar absorbers decreased the vaporization enthalpy and increased evaporation efficiency at the low solar intensities used in the desalination process and provided a means of detoxifying industrial effluent [45,55]. He et al. [41] demonstrated a method of constructing solar steam generators that is good for the environment. They used a pre-stretched electro-spun polyurethane (PU) nanofiber membrane as an elastic skeleton and photothermal materials such as CNTs and self-polymerized polydopamine (PDA) to make stable hierarchical nanostructures. Under weak sun intensities, the eco-friendly solar absorber could be used to purify oil-contaminated water with 90.1% efficiency. Furthermore, Yang et al. [56] developed a bio-inspired eco-friendly CNT system involving a sugarcane-coating-based steam generator under low solar flux, which showed an efficiency of 94.2% in purifying saline water. In addition to Equation (1) for lower solar radiation, it is necessary for such a system to absorb heat from its surroundings for real-world use [57]. Wang et al. [58] employed polystyrene foam to gather heat from the surroundings to raise the temperature of an MXene/CNT/cotton-based solar absorber. Under one-sun illumination, the solar steam generating system was shown to be suitable for purifying textile wastewater with 88.2% evaporation efficiency. A CNT-based solar absorber membrane exhibited high porosity, low thermal conductivity, and a high rate of solar light absorption as it absorbed additional energy from the environment. The essential parameters of CNT-based solar absorbers under weak solar irradiation are provided in Table S1.

In order to increase evaporator performance, Mu et al. [40] highlighted the need to match input energy (IE) with required energy (RE) under low sun intensity. They showed the rate of water transport in aerogels. They utilized natural wood to decrease the hydrophilicity of the CNT aerogel. The quantity of energy required was reduced to match the amount of energy available. This approach increased the rate of evaporation to 93.2%. The rate of evaporation rose by almost 40%. It was shown to have significant practical applications in desalination, oil-water separation, heavy metal ion treatment, and sewage treatment.

Due to the limited energy input provided by weak solar irradiation, energy consumption efficiency remains a significant issue for SWP, in addition to the previously described technique. Under low solar intensity, SWP can benefit from a unique structural design for energy capture. CNT-based three-dimensionally structured solar thermal absorbers display superior evaporation performance by minimizing energy losses and reflectivity or by increasing the surface area. The meticulously designed 3D structure can absorb visible light from multiple angles and utilize vast surfaces to harvest energy from the surroundings. The poor absorption rate in some wavelengths can be compensated for by designing a 3D structure to obtain the specific and selective absorption of sunlight. Using a 3D structure for SWP, it is possible to exceed the theoretical limit and provide practical applications for daily pure water production. Hong et al. [59] constructed a GO (graphene oxide)/CNT-based bio-inspired 3D origami-structured evaporator. Origami-based GO/CNTs are suitable for harsh environments and long-term use, such as salt elimination and antibacterial mechanisms. Wu et al. [60] also designed a biomimetic 3D-structured solar evaporation system with a 0.7 height-to-diameter ratio for each asymmetrical groove to enhance water transport. In the 3D evaporator developed by the Wu group, CNTs were used for the photothermal material, whereas sodium citrate particles were used to create the surface pores. Micropores were produced when sodium citrate was removed from the 3D structure. To filter saltwater and chemically contaminated water, an energy efficiency greater than 96% was achieved under a single solar illumination with remarkable stability, even under high salinity. Jinn et al. [61] reported on the development of a three-dimensional carrier material using bacterial cellulose (BC) combined with CNTs and rGO to generate composite sheets for solar evaporation. With a microporous cellulose matrix composite film, the rate of photothermal evaporation and the efficiency of photothermal conversion were increased. The photothermal conversion showed an efficiency of 90.2% and generated 23.32 kg/m^2^ of clean water from seawater during a single day of operation. CNT-based three-dimensionally organized solar absorbers can isolate heat and minimize heat loss. They capture solar energy from several angles and optimize efficiency beyond the theoretical limit. In order to produce CNT-based three-dimensionally structured absorbers on a large scale, additional studies are necessary due to the complexity of the materials prepared using three-dimensional structures. The key parameters of three-dimensionally structured CNT-based solar absorbers under weak solar irradiation are summarized in Table S2.

**Figure 5:**
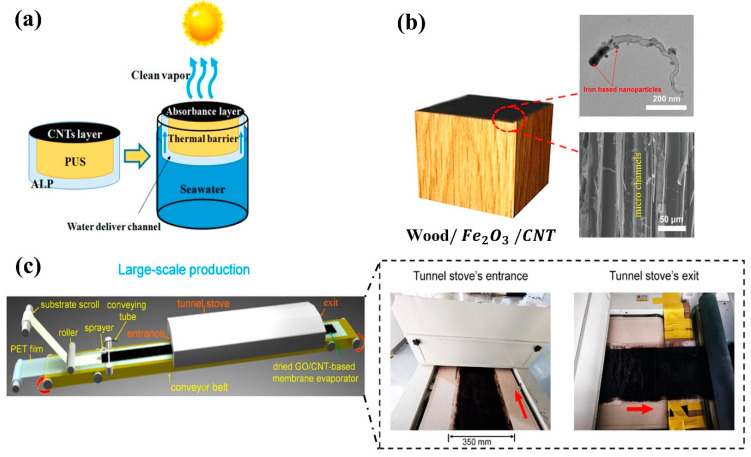
(**a**) Schematic of a solar thermal water purification system using a CNT-based floating solar still to collect high solar energy, PUS as a thermal isolator to preserve heat, and air-laid paper as a water transport channel to filter saltwater (reproduced with permission, Copyright 2019 American Chemical Society [62]. (**b**) Photograph and example of the use of capillary channels to reduce heat loss (reproduced with permission, Copyright 2021 American Chemical Society [67]). (**c**) Large-scale production of GO/CNTs as a solar absorber for solar thermal water purification (reproduced with permission, Copyright 2022 American Chemical Society [46]).

Obtaining irradiation from exactly one sun during large-scale practical operations is quite improbable. There are fundamental variations in solar irradiation and the quantity is often less than one sun, owing to weather, climate, and geographical location. A failure to consider this would result in difficulties in designing SWP devices, such as decreased evaporation efficiency, heat loss, inefficient water movement, and inadequate solar light absorption. The testing of novel SWP designs with only one-sun irradiation does not adequately depict how solar thermal water purification devices will perform in actual usage conditions. As demonstrated by Equation (1), increasing the energy input through solar energy collection, heat loss reduction, and water transportation is essential to boost the evaporation efficiency for large-scale clean water production, as shown in Figure 5a [43,62,63].

Researchers have shown interest in CNTs because of the problems listed above. Figure 3 shows that CNTs have been employed to fabricate a solar thermal water purification device, which was used as a high solar light absorber [49]. Jiang et al. [44] reported that a bilayer aerogel composed of CNTs and cellulose nanofibrils (CNFs) was a super-solar absorbing and thermally insulating material, demonstrating 81.3% evaporation efficiency under 3 sun irradiations ($1\;\mathrm{sun}\;=1\;\mathrm{kW}/m^{2}$). Yang et al. [64] developed an ultrathin 2D porous photothermal membrane based on SWCNT–MoS_2_ hybrid film with a thickness of 120 nm and a spectrum absorption more significant than 82%. Under 5 kW/m^2^ of solar light, the temperature reached 106 °C in a few seconds. The ultrathin 2D porous network structure of the SWNT–MoS_2_ film enabled rapid steam generation and minimized heat loss, resulting in 91.5% evaporation efficiency. Shi et al. [65] used magnetic force to separate the toxic chemical particles from the water. They prepared a solar heat absorber using Fe_3_O_4_@CNT. However, thermal efficiency dropped from 88.7% to 45.8% at 3 sun. At 10 sun, thermal efficiency was 84.9%, and part of the solar energy was used to heat the water directly. In contrast, the remainder was used to heat the surface nanoparticles, resulting in thermal efficiency losses in the surface nanoparticles. Furthermore, Wang et al. [47] also prepared 10 mg CNTs on 40-mm-diameter filter paper, by means of vacuum filtration, as a solar harvesting material. CNT films exhibited high capillary imbibition and wide absorption of solar light. The CNT sheet used solar energy to produce bio-inspired solar steam and evaporate the surrounding water molecules. Due to localized heating of the floating CNT film, water vapor rapidly escaped from the porous CNT film. The evaporation efficiency decreased from 54.6% to 45.3% as the intensity of solar light increased from $1\;\mathrm{kW}/m^{2}$ to $5\;\mathrm{kW}/m^{2}$. Previous investigations have indicated that CNT-based hybrid material utilized as a solar absorber has a lower evaporation efficiency when exposed to intense sunlight. The performance of CNT-based SWP devices has also been found to decrease under strong sunlight because of high capillary absorption and significant heat loss.

In addition to solar energy harvesting, a reduction in heat loss and increased water transport enhanced evaporation efficiency when using CNT-modified natural wood to maximize high solar energy absorption and minimize heat loss. The natural interconnected channels in the wood structure also maximized the transport of water to the solar absorbers for the production of clean water from groundwater [48] and seawater [66]. Li et al. [67] fabricated a bio-inspired solar thermal water purification system by heating and covering beach wood with Fe_2_O_3_/CNTs. The water flowed rapidly in an upward way through a porous wood layer that was vertically oriented, as shown in Figure 5b. The device achieved an evaporation efficiency of 99.8% under 10-sun illumination and successfully purified seawater and wastewater. Zhang et al. [46] presented a spray-based technique to mass-manufacture GO/CNT-based solar evaporator membranes. Figure 5c shows that they employed a tunnel to dry the membrane evaporator continuously. Nevertheless, experimental work has been conducted on a lab-scale GO/CNT solar absorber. Due to heat losses, they attained poor efficiency when increasing sun intensity. Table S3 presents the most important parameters governing the performance of CNTs-based solar absorbers under intense solar illumination.

### 2.2. Solar Thermoelectric Generator (STEG)

A solar thermoelectric generator (STEG) is an ecologically beneficial green energy technique, similar to photovoltaics. STEG has recently received research interest due to breakthroughs in thermoelectric material characteristics and STEG system design. It can be utilized in locations where other forms of energy generation are inapplicable because of their lack of moving components and ease of installation. Thermoelectric generators (TEGs) based on semiconductors are solid-state devices. In these systems, the Seebeck effect is utilized to directly convert thermal energy to electrical energy, which follows Equation (2):

(2) $$zT=\frac{S^{2}}{\kappa }\sigma T$$

where *zT* = figure of merit, *S* = Seebeck (${\mu \mathrm{VK}}^{-1}$) coefficient, κ = thermal conductivity (${\mathrm{Wm}}^{-1}K^{-1}$), σ = electrical conductivity (${S\;\mathrm{cm}}^{-1}$), and *T* = absolute temperature.

**Figure 6:**
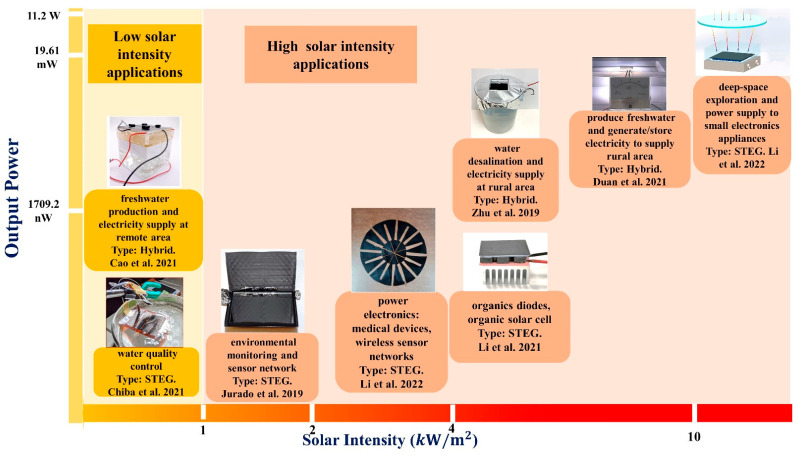
Application of CNT-based hybrid generators (water and electricity) (reproduced with permission, Copyright 2021 American Chemical Society [72]; reproduced with permission, Copyright 2019 John Wiley and Sons [73]; reproduced with permission, Copyright 2021 Elsevier [74]). CNT-based STEG (reproduced with permission, Copyright 2021, The Authors, Springer Nature [75]; reproduced with permission, Copyright 2019, The Authors, John Wiley and Sons [76]; reproduced with permission, Copyright 2022 Elsevier [77]; reproduced with permission, Copyright 2021 Elsevier [71]; reproduced with permission, Copyright 2019 Elsevier [78]).

The effectiveness of a thermoelectric device is influenced by its basic thermoelectric materials [68]. Bismuth telluride and lead telluride are two inorganic semiconducting materials that have drawn the most attention from the thermoelectric (TE) community because they are degenerate and have displayed excellent TE performance [69]. Degenerate inorganic semiconductors (bismuth, antimony, tellurium, selenium, and similar materials) rely on rare-earth metals, which are both costly and toxic, rendering them impossible to use for the manufacturing of large-area TE devices. In contrast, CNT-based organic TE devices are crucial due to CNTs’ excellent thermoelectric properties [70]. Researchers worldwide have exerted considerable efforts to raise the *zT* value of CNT-based STEG [71]. Figure 6 illustrates the utilization of CNT solar thermoelectric generators in individual and hybrid applications at high and low solar intensities.

Researchers have used CNTs in two ways: either as a solar absorber placed over the hot side of the thermoelectric modules or as a component in the construction of thermoelectric modules [71,79,80]. Both uses helped the researchers to achieve high-temperature differences and high output powers.

Xia et al. [81] fabricated a thermoelectric generation device based on bismuth telluride and CNTs. The elements (128 units of Bi_2_Te_3_ p-type and n-type) were connected in series, and CNT sheets were used to cover the device’s top surface. The integrated device used CNT sheets to absorb solar heat and Bi_2_Te_3_ to generate thermoelectric electricity. The layers of CNT sheets were overlapped crosswise to produce CNT sheets with an areal density of 0.32 g/m^2^. Crosswise-overlapping CNT sheets increased light absorption to 95%. One hundred twenty-eight Bi_2_Te_3_ units were used to build the device, which was 4.8 cm × 4.8 cm in size and covered with 0.32 g/m^2^ CNT sheets. They obtained an open circuit voltage (V_oc_) of 400 mV and a short circuit current density (J_sc_) of 5520 mA/cm^2^ without an optical or thermal concentrator in the near-infrared field. In addition, they achieved a power conversion efficiency of 2.1%. They concluded that this integrated device was long-lasting, stable, and capable of operating at high temperatures. However, additional research is required for large-scale operation. In addition, Li et al. [78] designed and constructed a solar thermoelectric generator that incorporated a solar concentrator and a CNT solar absorber. Bi_2_Te_3_ thermoelectric modules were used to generate electricity. They applied the MWCNT sunlight absorbent layer on the hot side of the TE module to improve thermal conversion efficiency. They placed a solar simulator on top of the TEG module and a Fresnel lens for sunlight concentration beneath it. The device attained its maximum voltage of 11.6 V and its maximum power of 11.2 W with a temperature difference of 178 °C under intense solar radiation. Furthermore, the device’s performance was evaluated, along with convection and radiative heat losses from TE modules, and a CNT layer of more than 0.5 g/m^2^ led to increased thermal losses.

Jurado et al. [76] demonstrated the capabilities of organic thermoelectric materials such as CNTs to gather solar energy in a STEG system. They utilized cobalt molybdenum catalytic method (CoMoCat) CNTs and an enhanced direct-injection pyrolytic synthesis (eDIP)-cellulose CNT composite to collect incoming solar radiation and then transport the concentrated heat to the legs of TEGs via a substrate with a high thermal concentration. Ten milligrams of CNTs were mixed in a 50 mm aqueous solution with a 1 mg/mL sodium dodecylbenzene sulfonate concentration (SDBS). These dispersions were sonicated using a bath sonicator before being centrifuged. Then, a filter with a 2 μm pore size was used to achieve 10-μm-thick Bucky paper with 10 wt% CNTs. Finally, The STEG device with 6 legs reached 180 nW under 2-sun illumination, powering small sensors for IoT devices and environmental monitoring.

Chiba et al. [75] used only p-type SWCNT films to make and test TEGs that float on water. They prepared SWCNTs using the vacuum filtration method. They utilized a polyimide sheet as the substrate and drilled four holes for the SWCNTs components. The SWCNT films were linked together in series with tiny copper wires. One end of the film was connected to a heat sink, and the other end was connected to a heater. They floated an SWCNT-based TEG device on water and applied wind to the TEGs to investigate the device’s various configurations. The output power of the TEGs was measured after they were irradiated with an artificial sun simulator to replicate direct sunlight. Under one sun, the device achieved an output voltage of 1300 µV and an output power of 22.8 nW at a water temperature of 80 °C and a wind speed of 3 m/s. They stated that additional research was required to optimize the size of SWCNT films to boost voltage and power.

Wu et al. [36] developed an all-solid-state flexible thermoelectric generator (AF-TEG) for low-power electronics. They employed thin-film MWCNTs and created the device in three stages. They dried n-type MWCNTs after soaking them in polyethyleneimine (PEI) solution. In the 1st stage, they passed p-MWCNT films through FeCl_3_ and dried them for 12 h. Then, p-type and n-type MWCNT films were sliced into rectangles. In the 2nd stage, TEG films were made by hot pressing multilayered p-type and n-type functionalized films. Polyvinylidene fluoride (PVDF) was used to avoid electrical contact. In the 3rd stage, 10 TE films were coupled in series with silver wires and secured in copper foil and soft polyimide sheets. In the experiment, convex lenses focused sunlight to heat the hot ends of the MWCNT films. They reached the conclusion that the AF-TEG device was applicable in waste heat harvesting and wearable electronics.

Zhang et al. [82] developed an innovative light-driven flexible STEG system, with a vanadium dioxide (VO_2_) flexible film to regulate light and a CNT-based flexible thermoelectric device to absorb light and convert it to photo-thermoelectric energy. They made flexible p-type and n-type thermoelectric films and modules using SWCNTs, polyvinylidene fluoride (PVDF) as the raw material, and polyethyleneimine (PEI) as an electron donor. They applied VO_2_ flexible material on the hot side to intelligently manage solar light. According to the design of their device, it could be folded in half without degrading any fluctuations. On the cold side, aluminum foil was used to prevent light absorption. They constructed the TEG module with six layers to make it more flexible. Experiments showed that the system could produce a stable 6.4 mV when exposed to natural sunlight. The researchers found that the manufactured device was flexible, robust, and had a steady voltage output.

**Figure 7:**
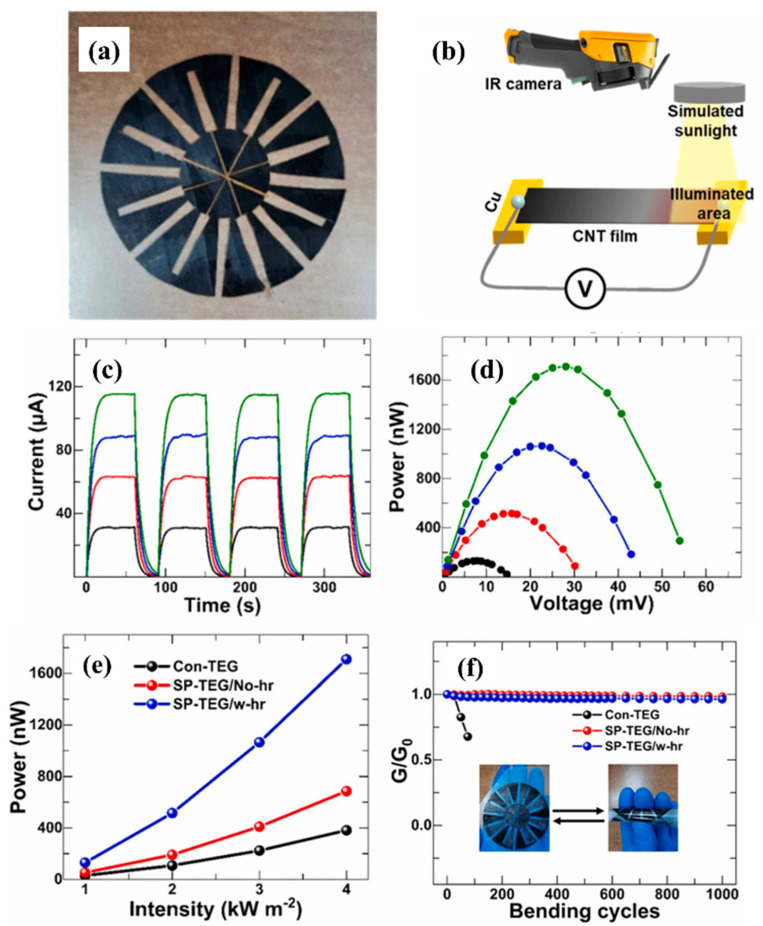
(**a**) Image of a flexible TEG. (**b**) Schematic presentation of the experimental setup. (**c**) The output current response and (**d**) output power-voltage curve of the TEG at different intensities (black, red, blue and green color represents $1\;\mathrm{kW}/m^{2}$, $2\;\mathrm{kW}/m^{2}$, $3\;\mathrm{kW}/m^{2}$ and $4\;\mathrm{kW}/m^{2}$ respectively) (**e**) The maximum output power curve of the Con-TEG and SP-TEG. (**f**) Bending test of the SP-TEG and Con-TEG (reproduced with permission, Copyright 2022 Elsevier [77]).

Recently, Li et al. [77] developed an organic solar thermoelectric generator (SP-TEG) utilizing heat rectifying junction-free trapezoidal structured p/n modules and CNT films as thermal conversion materials as shown in Figure 7a. Experiments were conducted to measure the performance of CNT films based on their solar thermoelectric characteristics. CNT film strips of 30 mm in length and 5 mm in width were attached between two copper electrodes. As illustrated in Figure 7b, sunlight irradiated one side of the CNT film, and an IR camera captured the generated voltage. Figure 7c shows that the current response to time exhibited a rising pattern, whereas Figure 7d shows an ascending power-voltage curve. Both figures were derived from the SP-TEG experiments shown in Figure 7b. These graphs were generated as the intensity of solar light grew from 1 to 4 suns. Due to the low internal resistance, the CNT-film TEG demonstrated a linear relationship with sunlight and increased voltage. The output power was calculated using the equation described by Liu et al. [79]. This device generated 1709.2 nW power under 4 suns under the same ambient conditions. The maximum output power under the abovementioned conditions increased quadratically with increasing light intensity, as shown in Figure 7e. In addition to their light-to-electricity conversion capabilities, the MWCNT-based TEGs demonstrated remarkable bending qualities. The conductivity of the flexible devices was unchanged even after one thousand bending cycles, as shown in Figure 7f. Table S4 summarizes the critical parameters that affect the performance of CNT-based solar thermoelectric generators when exposed to solar irradiation.

**Figure 8:**
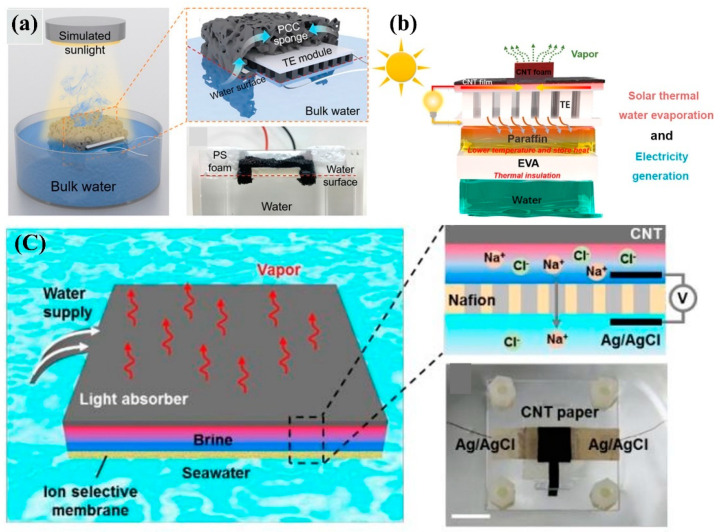
(**a**) Schematic diagram of the successive generation of water and steam using a CNT composite sponge as a solar absorber (reproduced with permission, Copyright 2019 John Wiley and Sons [73]). (**b**) Illustration of CNT/TE/paraffin/EVA/water hybrid structure steam and electricity generation (reproduced with permission, Copyright 2021 American Chemical Society [72]). (**c**) Mechanism of the hybrid device for water purification and electricity generation from salinity gradient (reproduced with permission, Copyright 2017 The Royal Society of Chemistry [86]).

In recent years, researchers have examined a more feasible method of producing energy from the evaporation of water, utilizing the latent heat of vapor. The significant variation in temperature serves as an energy source [83]. CNTs with rough surfaces and porous structures exhibit high sunlight absorption, high electrical conductivity, and outstanding energy storage capability, making them appropriate for water purification and electricity generation [74]. Figure 6 shows that the CNT-based hybrid system produced more energy at the same solar intensity than the thermoelectric generator alone. The most plausible reason for this is that water reduces the temperature on the cold side. CNT solar absorbers store solar heat on the hot side and generate thermoelectric power on the basis of the significant temperature differential. In 2019, Zhu et al. [73] fabricated a CNT/CNC nanocomposite-based 3D Bucky sponge, which could generate electricity in addition to performing water purification by localizing heat and recycling steam enthalpy, as shown in Figure 8a. The TE module was covered by the sponge to achieve a high-temperature difference under 5-sun illumination. The open-circuit voltage, short-circuit current, and output power were observed to be 2.63 mV, 110 mA, and 5.38 mW, respectively. Ding et al. [84] developed a natural CNT/wood composite nanogenerator for water purification and thermoelectric generation. Under a single sun, the addition of Fe mesh to the CNT/wood structure produced a power density of 0.35 mW/m^2^ in deionized water. Cao et al. [72] developed a hybrid CNT-based system that can generate both potable water and power. In the core, both the hydrophobic CNT film and the hydrophilic CNT foam/PVA serve as heaters and evaporators, respectively. The temperature gradient between the CNT film and the paraffin layer led to the temperature variance of the TE module, as shown in Figure 8b. The CNT film/TE/paraffin/CE system obtained an open-circuit voltage, short-circuit current density, maximum power density, and output power density of 96.35 mV, 1.65 mA/cm^2^, 0.38 mW, and 0.4 W/m^2^, respectively, with a load resistance of 6 ohm.

Instead of reusing the latent heat, the constant generation of steam can have other effects, such as modifying the salt concentration [85]. Yang et al. [86] proposed that saline water could be utilized uniquely to generate energy, as shown in Figure 8c. In his study, CNT-modified paper was utilized in the hybrid device’s top layer, and an ion-selective membrane, such as Nafion, was employed in the device’s bottom layer. It was observed that salt ions were directly transferred from the evaporator to the bulk seawater, resulting in the formation of a salinity gradient [87]. Under 2-sun illumination, solar power generation had an efficiency of 0.6%, resulting in an output density of 1 W/cm^2^, and a saturated voltage of 84 mV. The experimental results concerning CNT-based solar hybrid generators and the key factors influencing their performance are summarized in Table S5.

### 2.3. Solar Water Heater

**Figure 9:**
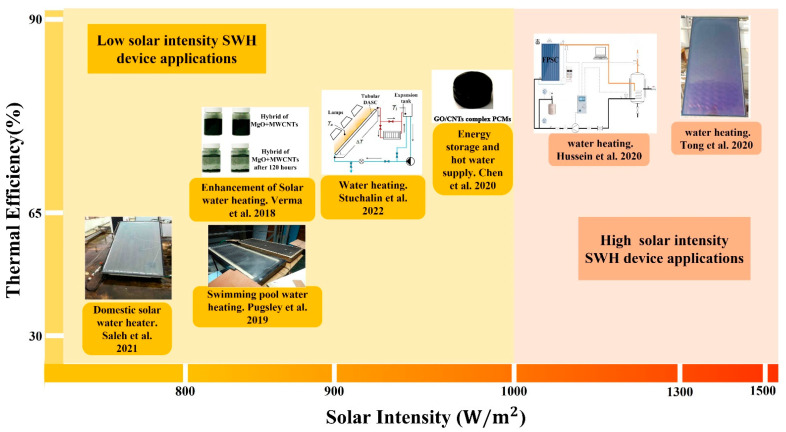
Application of CNT-based solar water heating (SWH) devices (reproduced with permission, Copyright 2021 MDPI [92]; reproduced with permission, Copyright 2019 Elsevier [93]; reproduced with permission, Copyright 2018 Elsevier [94]; reproduced with permission, Copyright 2022 MDPI [95]; reproduced with permission, Copyright 2020 Elsevier [96]; reproduced with permission, Copyright 2020 Elsevier [97]; reproduced with permission, Copyright 2020 Elsevier [98]).

Solar water heating (SWH) is a prevalent household and industrial heating technology [88]. A solar water heater is a device that heats water using the sun’s light energy. The SWH system is one of the most efficient types of solar thermal collectors within the domain of renewable energy technology. SWH systems are gaining popularity due to their low cost, low environmental impact, and long lifespan. Researchers have improved various components and pieces of equipment to increase the performance of solar collectors as part of the substantial effort being made to meet sustainable development goals. The capability of nanofluids to absorb sunlight has garnered considerable attention. The adoption of CNT nanofluids rather than conventional fluids is currently the most effective approach for increasing solar collectors’ thermal efficiency [89,90]. Figure 9 illustrates how solar thermal collectors are used to heat water under different solar intensities. In this section, we discuss broad and in-depth studies of the thermal efficiency and applications of solar thermal collectors used to heat water at high and low solar intensities. The following equation is used to derive the thermal efficiency of the solar collector [91]:

(3) $$\eta =\frac{Q_{u}}{I_{t}A_{c}}\;\left(\%\right)$$

where η = thermal efficiency, $Q_{u}$ = input energy, $I_{t}$ = solar intensity, and $A_{c}$ = area of the solar collector.

Solar collectors that use water as the primary working fluid are frequently employed to heat water [99]. In terms of thermal efficiency and solar energy absorption, however, these systems do not perform as well as expected. Nanofluids based on carbon nanotubes have been optimized for solar light absorption and have reached maximum efficiency [100,101,102]

Numerous studies have utilized nanofluids based on CNTs for heat transfer and water heating in solar collectors under weak (1000 W/m^2^) and high (>1000 W/m^2^) solar intensities. Under low solar intensity, Ghodbane et al. [103] reported that a linear Fresnel solar collector with a 0.3% vol concentration of industrial-grade MWCNT/DW nanofluid exhibited a thermal efficiency of 33.81%. Kim et al. [104] obtained 57.4% thermal efficiency under 800 W/m^2^ solar intensity by experimentally combining 2 wt% CNTs with the twin-walled polycarbonate absorber of a flat plate solar collector. Using the same experimental conditions, Pugsley et al. [93] evaluated flat plate solar collectors (FPCs) and evacuated tube solar collectors (ETSCs) and measured a thermal efficiency of 62%. In addition, they reduced the size of the collector and utilized it to heat swimming pool water. Mahbubul et al. [105] examined the efficiency of an evacuated tube solar collector including SWCNTs as the working nanofluid. Under 900 W/m^2^, the water temperature reached 120 °C and the thermal efficiency reached 66.7%. Eltaweel et al. [106] examined the effect of MWCNT/water nanofluid as a working fluid in a flat-plate solar collector with a flow rate of 1.5 L/min and a weight fraction of 0.1%. They found that increasing solar intensity enhanced thermal efficiency, with a peak thermal efficiency of 70.67% attained under a solar irradiance of 915 W/m^2^. Verma et al. [107] investigated the thermal efficiency performance of six different nanofluids applied to a flat-plate solar water heater. At a volume concentration of 0.2 wt% and a reasonably high solar intensity, the thermal efficiency of MWCNT/water nanofluid was significantly improved. Similarly, Tong et al. [98] analyzed the performance of FPCs by comparing the sensitivities of numerous nanofluids under a diverse range of conditions. The nanofluid containing MWCNTs with a particle size of 20 nm was found to have the highest solar thermal efficiency of 87% when exposed to a solar intensity of 1500 W/m^2^. Based on the above experimental results, CNT-based nanofluid has proven to be an excellent alternative to use in place of water as the working fluid in SWH to achieve high thermal efficiency.

Nonetheless, according to a review of the relevant research, hybrid nanofluids would be superior to mono nanofluids in thermal systems under different intensities of solar power [108,109,110,111]. Saleh et al. [92] examined the performance of flat-plate solar water heaters using hybrid MWCNT + Fe_3_O_4_/water nanofluids at a volume concentration of 0.3. They achieved 63.85% thermal efficiency by employing 785 W/m^2^ of solar radiation. Verma et al. [94] examined the performance of two distinct nanofluid hybrids. They maintained the same collector size but altered the nature of the material. After conducting experiments using a Cu/MWCNT hybrid fluid and an MgO/MWCNT hybrid nanofluid, the researchers found that the MgO/MWCNT hybrid nanofluid reached 70.55% efficiency under 800 W/m^2^ solar radiation. Mashhadian et al. [112] evaluated the environmental effects of the direct absorption of parabolic-trough solar collectors when weak solar radiation was present. They prepared an Al_2_O_3_ + MWCNTs hybrid nanofluid dispersed in water with a 0.04 wt% concentration. They found that under a solar intensity of 856 W/m^2^, the thermal efficiency could be increased to 64.9% while simultaneously decreasing CO_2_ emissions. Struchalin et al. [95] conducted experimental research on the thermal performance of hybrid Fe_3_O_4_/MWCNT nanofluids for direct absorption solar thermal collector (DASC) applications. The hybrid nanofluid enhanced the temperature gradient of domestic hot water, and the DASC utilizing the hybrid nanofluids displayed a thermal efficiency of 69.4% at 915 W/m^2^ ± 10%. The DASC utilizing the MWCNT nanofluid demonstrated better thermal efficiency than the DASC using a magnetic + MWCNT hybrid nanofluid. Hussein et al. [97] synthesized triple-hybrid nanofluids including covalent functionalized multi-wall carbon nanotubes (CFMWCNTs), covalent functionalized graphene nanoplatelets (CFGNPs), and hexagonal boron nitride in FPSC (h-BN). Under a solar intensity of 1300 W/m^2^, the thermal efficiency of FPSC at a volumetric flow rate of 4 L/min climbed to 85%.

Researchers have recently concentrated on developing phase change materials (PCMs) based on CNTs for use in solar water heating technologies in order to improve the thermal efficiency of solar water heaters [113,114]. Sobhansarbandi et al. [115] enhanced the absorptivity of vacuum tube solar collectors (ETCs) for water heaters by employing “dry-drawable” CNT plate coatings. The solar collector utilized PCM, octadecane paraffin with a melting point of 28 °C and a heat fusion of 244 kJ/kg. The results showed that 15 was the optimal number of layers, and that the ability of coated glass to absorb 947 W/m^2^ of solar radiation was increased by up to 98%. When maintaining water temperature using PCMs in solar water heaters, the water temperature is relatively constant during the day. Chamkha et al. [116] investigated the impact of using MWCNTs and paraffin as PCMs in a solar still for hot water generation. The thermal properties of PCMs were improved by mixing MWCNTs into paraffin wax. The researchers increased the rate of hot water production by 19.6% using PCM, while obtaining a thermal efficiency of 58.7% at a solar intensity of 1010 W/m^2^. Chen et al. [96] prepared a PCM comprising paraffin wax, graphene aerogel, and carbon nanotubes using a hydrothermal technique. The thermal performance of the solar water heater was determined to be 73% at a solar intensity of 1000 W/m^2^. The experimental results concerning CNT-based solar water heaters are summarized in Table S6, including the factors that affect the performance of the devices.

## 3. Conclusions

In this study, we analyzed the current development and application of CNTs in solar thermal systems, operating under varying levels of solar irradiance. This comprehensive literature evaluation has yielded information on several research paths, gaps, and outcomes. It has highlighted the current efforts of researchers to improve the effectiveness of water purification, thermoelectric production, and water heating systems using CNTs under low and high sun intensities. In this review, we have also focused on the many indicators and technological advancements that have occurred in water purification, thermoelectric production, and water heating systems using encapsulated CNTs, which significantly improve overall performance. The following can be determined after a comprehensive review of previous articles.

- CNTs are the optimal material for photothermal conversion devices because they are black-body absorbers with high solar light absorption and minimal infrared emissions.
- Applying CNT composites and CNT coatings in solar water purification devices and solar thermoelectric generation devices leads to a remarkable enhancement in the system’s overall efficiency.
- Aerogels composited with CNTs have a soft, porous structure that allows them to function as a secondary filter, eliminating microscopic particles and harmful compounds that generally persist in contaminated water.
- The addition of CNTs to the p/n module of TEGs enhanced the devices’ overall performance by increasing the temperature gradient required to generate electricity.
- CNTs have a high capacity for absorbing heat. The absorber receives diffused solar radiation due to large and small molecules in the environment. CNT-based thermoelectric devices have been shown to produce more power when employing an optical concentrator.
- The thermal storage properties of CNT-based nanofluids increased the collection efficiency of SWH, keeping water hot for an even more prolonged period.
- CNTs with strong hydrophobic properties facilitate the transmission of heat to water without interacting with water molecules.

## 4. Open Issues to Be Developed

The development of solar thermal devices based on CNTs is currently confronted with a range of issues. Fundamental research on solar thermal devices has a long way to go. Too little consideration has been given to how solar radiation affects thermal equipment. The following is a list of potential avenues of study uncovered in this review:

1. Due to their nano-size and the difficulty involved in isolating and manipulating them, the practical applications of CNTs for water filtration are restricted. The removal of such microscopic particles from large quantities of water incurs additional costs. Cost-effectively increasing the thermal utilization efficiency of CNT-based photothermal materials remains a substantial obstacle.
2. Numerous techniques, including arc discharge, chemical vapor deposition, laser ablation, and flame synthesis, have been devised to produce CNTs. Most of them are time-consuming and involve complex equipment and processes. Another significant problem is that the CNTs synthesized via the proposed techniques are highly hydrophobic, preventing the effective transfer of water to the evaporating surface.
3. It is not yet possible to achieve CNT growth across a large space. The poor performance of water purification systems and the absence of technologies for their production are barriers to their use on a large scale.
4. Natural solar radiation can be reduced in strength due to the influence of many factors, including weather, climate, and geographical circumstances. Because of the unpredictable nature of solar irradiation, relying solely on testing procedures conducted in the laboratory is a very impractical approach.
5. CNT-based SWP devices subjected to intense solar irradiation showed an increase in heat losses due to the significant size of the energy input. It is necessary to perform further research in order to reduce the amount of heat lost due to convection, radiation, and conduction.
6. STEG systems still operate with low overall efficiency. Additional research has to be conducted on CNT-based systems encapsulating absorbing materials such CNT coatings and CNT composite materials to improve the overall efficiency of TEGs. These are both implemented on the hot side of the TEG.
7. According to the research, only a small number of studies have been performed to investigate how the level of sun intensity affects solar water heating systems. It is recommended that high solar power be implemented over the solar collector in industrial applications in order to generate a higher level of heat energy and produce a large quantity of hot water.
8. CNT-based hybrid nanofluids provide a number of benefits over conventional ones. No definitive research has been conducted on the viability of the use of hybrid nanofluids containing CNTs in solar water heating systems. MWCNT nanofluid has attained a high level of effectiveness to date. However, its preparation costs are significantly higher than those of hybrid nanofluids. Consequently, more studies are necessary in order to fabricate hybrid nanofluids based on CNTs.
9. It has been shown that CNT-based PCMs for solar water heating systems have relatively few applications, which means that further research must be carried out in the near future.

## Data Availability

All data generated or analyzed during this study are included in this published article and its Supplementary File.

## Supplementary Materials

The following supporting information can be downloaded at: https://www.mdpi.com/article/10.3390/nano12213871/s1, Table S1: Summary of the key parameters for CNT-based SWP under weak solar irradiation; Table S2: Key parameters of three-dimensionally structured CNT-based solar absorbers; Table S3: Key parameters of CNT-based solar absorbers for water purification under strong solar intensity; Table S4: Key parameters of CNT-based solar thermoelectric generators; Table S5: Key parameters of CNT-based solar hybrid generators; Table S6: Key parameters of CNT-based solar water heaters.
