# Supplementary material for: Recent Development of Carbon-Nanotube-Based Solar Heat Absorption Devices and Their Application
Source: Nanomaterials (Basel). 2022 Nov 2;12(21):3871. doi: 10.3390/nano12213871 (PMC9658299; doi:10.3390/nano12213871)
Supplement: Supplementary file 1 [file nanomaterials-12-03871-s001.zip › nanomaterials-1985584-supplementary.pdf]

nanomaterials

MDPI

Supplementary Materials

Recent development of Carbon nanotubes based solar heat absorption- devices and their application

Saiful Islam1,2* and Hiroshi Furuta1,2*

[1] School of Systems Engineering, Kochi University of Technology, Kochi 782-8502, Japan
[2] Center for Nanotechnology, Research Institute, Kochi University of Technology, Kochi 782-8502, Japan
* Correspondence: 256003q@gs.kochi-tech.ac.jp (S.I.); furuta.hiroshi@kochi-tech.ac.jp (H.F.);
Tel.: +81-887-57-2211

Table S1. Summary of the key parameters for the CNT based SWP under weak solar irradiation

Title	Year	Materials used	Solar intensity (kWm^{-2})	Evaporation rate ($kg\ m^{-2}h^{-1}$)	Evaporation efficiency	Ref.
Superwetting Monolithic Hollow-Carbon-Nanotubes Aerogels with Hierarchically Nanoporous Structure for Efficient Solar Steam Generation	Mu et al. (2018)	CMP based carbon aerogel	1	1.446	86.8%	51
Enhanced solar steam generation using carbon nanotube membrane distillation device with heat localization	Miao et al. (2018)	CNT membrane,	1	1.31	84.6%	45
Flexible and Washable CNT-Embedded PAN Nonwoven Fabrics for Solar-Enabled Evaporation and Desalination of Seawater.	Zhu et al. (2019)	polyacrylonitrile (PAN) and CNTs	1	1.44	81%	42
Self-floating aerogel composed of carbon nanotubes and ultralong hydroxyapatite nanowires for highly efficient solar energy-assisted water purification	Qin et al. (2019)	Hydroxyapatite (HAP) nanowire aerogel and CNTs	1	1.34	89.4%	37
Energy Matching for Boosting Water Evaporation in Direct Solar Steam Generation	Mu et al. (2020)	bilayer-structures of carbon nanotubes aerogel (CA)-coated wood (CACW)	1	2.22	93.2%	40
Highly Efficient Solar Steam Generation under Low Solar Flux via Carbon-Nanotube-Modified Sugarcane	Yang et al. (2021)	CNT-sugarcane bilayer structure	1	1.63	94.2%	56
One-step fabrication of a stretchable and anti-oil-	He et al. (2021)	Polydopamine-encapsulated	1	1.44	90.1%	41

fouling nanofiber membrane for solar steam generation		carbon nanotube/polyurethane (PDA@CNT/PU) nanofiber membrane				
Gradient Heating Effect Modulated by Hydrophobic/Hydrophilic Carbon Nanotube Network Structures for Ultrafast Solar Steam Generation	Cao et al. (2021)	Hydrophobic CNT film (Heating layer) and hydrophilic polyvinyl alcohol (PVA)/CNT foam (evaporating layer)	1	4.2	—	57
Simple and robust MXene/carbon nanotubes/cotton fabrics for textile wastewater purification via solar-driven interfacial water evaporation	Wang et al. (2021)	MXene/CNTs/cotton fabrics	1	1.35	88.2%	58
Synergy of photothermal effect in integrated 0D Ti2O3 nanoparticles/1D carboxylated carbon nanotubes for multifunctional water purification	Li et al. (2022)	PDA@Ti_2O_3 NPs and CNTs	1	1.81	92.4%	52
Carbon-supported nano tungsten bronze aerogels with synergistically enhanced photothermal conversion performance: Fabrication and application in solar evaporation	Li et al. (2022)	rGC-CWO/corn straw	1	1.93	85.9%	54
High-Performance Freshwater Harvesting System by Coupling Solar Desalination and Fog Collection with Hierarchical Porous Microneedle Arrays	Zhou et al. (2022)	polyethylene glycol diacrylate (PEGDA)/ Sodium alginate (SA)/CNT (MNPSC)	1	2.46	91.14%	50
Asymmetric Cellulose/Carbon Nanotubes Membrane with Interconnected Pores Fabricated by Droplet Method for Solar-Driven Interfacial Evaporation and Desalination	Yang et al. (2022)	Asymmetric Cellulose/CNTs membrane	1	1.6	89%	55

Table S2. Key parameters of three-dimensional structured CNT based solar absorbers

**Title**	**Year**	**Materials used**	**Solar intensity (kWm^{-2})**	**Evaporation rate ($kg\ m^{-2}h^{-1}$)**	**Evaporation efficiency**	**Ref.**
Nature-Inspired, 3D Origami Solar Steam Generator toward Near Full Utilization of Solar Energy	Hong et al. (2018)	Graphene oxide/CNT composite	1	1.59	>85%	59
Highly efficient three-dimensional solar evaporator for high salinity desalination by localized crystallization	Wu et al. (2020)	Nano composite of CNTs and citrate sodium	1	2.63	>96%	60
Hierarchically Designed Three-Dimensional Composite Structure on a Cellulose-Based Solar Steam Generator	Jin et al. (2022)	Bacterial Cellulose (BC)/ reduced Graphene oxide(RGO)/CNTs	1	1.85	90.2%	61

Table S3. Key parameters of CNT based solar absorbers for water purification under strong solar intensity

**Name**	**Year**	**Materials used**	**Solar Intensity (kWm^{-2})**	**Evaporation rate ($kg\ m^{-2}h^{-1}$)**	**Evaporation efficiency**	**Ref.**
Lightweight, Mesoporous, and Highly Absorptive All-Nanofiber Aerogel for Efficient Solar Steam Generation	Jiag et al. (2017)	Cellulose nanofibril (CNF)-CNT bilayer aerogel	1	1.11	76.3%	44
			3	3.52	81.4%	
Simple, Low-Dose, Durable, and Carbon-Nanotube-Based Floating Solar Still for Efficient Desalination and Purification	Gan et al. (2019)	1) MWCNTs 2) Air-laid paper with BET surface area 3) Polyurethane sponge (PUS) Name: ALP-CNTs -5mg	2	1.58	—	62
Flame Synthesis of Superhydrophilic Carbon Nanotubes/Ni Foam Decorated with Fe2O3 Nanoparticles for Water Purification via Solar Steam Generation	Han et al. (2020)	Fe_2O_3/CNT/Ni Foam nanocomposite	2	3	91.3%	43
			3	4.27	93.8%	
Coating of Wood with Fe2O3-Decorated Carbon Nanotubes by One Step Combustion for Efficient Solar Steam Generation	Li et al. (2021)	Beech wood, Ferric acetylacetonate, CNT	10	14.01	—	67

An Ultrathin Flexible 2D Membrane Based on Single-Walled Nanotube–MoS2 Hybrid Film for High Performance Solar Steam Generation	Yang et al. (2017)	SWNT-MoS_2 film	5	6.6	91.5%	64
Highly Flexible and Efficient Solar Steam Generation Device	Chen et al. 2017	CNT coated Balsa wood	10	11.22	81%	66
Extremely Black Vertically Aligned Carbon Nanotube Arrays for Solar Steam Generation	Yin et al. 2017	VACNTs array	15	10	90%	49
Enhanced direct steam generation via a bio-inspired solar heating method using carbon nanotube films	Wang et al. (2017)	CNT films	5	3.615	40%	47
Recyclable Fe3O4@CNT nanoparticles for high-efficiency solar vapor Generation	Shi et al. (2017)	CNTs with magnetic Fe_3O_4(Fe_3O_4@CNT)	1	—	43.8%	65
			3	—	23.3%	
			10	—	60.32%	
All Natural, High Efficient Groundwater Extraction via Solar Steam/Vapor Generation	Wang et al. (2018)	CNTs	10	12	86%	48
Multiscale Preparation of Graphene Oxide/Carbon Nanotube-Based Membrane Evaporators by a Spray Method for Efficient Solar Steam Generation	Zhang et al. (2022)	Graphene oxide (GO)/CNT	5	4.3	70.5%	46

Table S4. Key parameters of CNT based Solar thermoelectric generators

Title	Year	Materials Used	Solar intensity	Temp. difference	Voltage	Power	Ref.
A demo solar thermoelectric conversion device based on Bi2Te3 and carbon nanotubes	Xia et al. (2015)	Bi_2Te_3 module and CNTs used as a cover	110 $mWcm^{-2}$	—	400 mV	—	81
Combined solar concentration and carbon nanotube absorber for high performance solar thermoelectric generators	Li et al. (2018)	customized Be_2Te_3 modules and CNTs absorber used in hot side	6 kWm^{-2} to 200 kWm^{-2}	170 °C	11.6 V	11.2 W	78
Solar Harvesting: a Unique Opportunity for Organic Thermoelectrics?	Jurado et al. (2019)	CoMoCat CNTs eDIPS CNT composite	2 kWm^{-2}	30K	—	180 nW	76

Heat source free water floating carbon nanotube thermoelectric generator	Chiba et al. (2021)	SWCNTs	1 kWm^{-2}	80 °C	1300 μV	22.8 nW	75
Intelligent light-driven flexible solar thermoelectric system	Zhang et al. (2021)	SWCNT,polyvinylide fluoride (PVDF) thermoelectric module	100 $mWcm^{-2}$	50K	6.4 mV	65.7 nW	82
Enhanced thermoelectric performance and tunable polarity in 2D Cu2S-phenol superlattices composites for solar energy conversion	Li et al. (2021)	p-type CPSL/CNT30 and n-type CP-SL/PEI-CNT30 4 pair TE module	5 kWm^{-2}	—	6.86 mV	534.7 nW	71
Novel Wearable Pyrothermoelectric Hybrid Generator for Solar Energy Harvesting	Zhang et al. (2022)	CNT/CT solar absorber	1.5 kWm^{-2}	—	3.7V	—	80
All-in-one single-piece flexible solar thermoelectric generator with scissored heat rectifying p-n modules	Li et al. (2022)	MWCNTs	4 kWm^{-2}	70.9 °C	3.57 mV per leg	1709.2 nW	77

Table S5. Key parameters of CNT based Solar hybrid generators

**Title**	**Year**	**Materials Used**	**Solar intensity**	**Voltage**	**Power density**	**Power**	**Ref.**
Solar-driven simultaneous steam production and electricity generation from salinity	Yang et al. (2017)	CNT modified filter paper as light absorber Nafion membrane used as the ion selective membrane and Ag/AgCl as the electrode system	Natural solar power (avg solar flux 0.7 kWm^{-2})	66 mV	—	3 mW	86
Exploring Interface Confined Water Flow and Evaporation Enables Solar-Thermal Electro Integration Towards Clean Water and Electricity Harvest via Asymmetric Functionalization Strategy	Xiao et al. (2019)	CNTs film on modified PDMS filter paper	1 kWm^{-2}	0.55 V under 1 G ohm load resistance	—	2.1 μW	85
Shape Conformal and Thermal Insulative Organic Solar	Zhu et al. (2019)	Bucky sponge (CNT/cellulose nanocrystals composite on	5 kWm^{-2}	—	—	5.38 mW	73

Absorber Sponge for Photothermal Water Evaporation and Thermoelectric Power Generation		a polydimethylsiloxane (PDMS) sponge) Covered the conventional TE module by bucky sponge					
Carbon Nanotube Network-Based Solar-Thermal Water Evaporator and Thermoelectric Module for Electricity Generation	Cao et al. (2021)	CNT film covers the TE module	1 kWm^{-2}	—	1.1 Wm^{-2}	0.38 mW	72
CNTs/Wood Composite Nanogenerator for Producing Both Steam and Electricity	Ding et al. (2021)	MWCNTs modified woodblock	1 kWm^{-2}	—	0.35 mWm^{-2}	—	84

Table S6. Key parameters of CNT based solar water heaters

**Title**	**Year**	**Materials Used**	**Solar collector type and size**	**Solar intensity (Wm^{-2})**	**Thermal efficiency**	**Ref.**
Experimental evaluation of flat plate solar collector using nanofluids	Verma et al. (2017)	0.2% MWCNT/base fluid water	FPC	1300	23.47% enhancement compared with water	107
Evacuated tube solar collector with multifunctional absorber layers	Sobhansarbandi et al. (2017)	‘dry-drawable” Carbon Nanotube (CNT) sheet coating 15 layer densified MWCNT + 14g paraffin	Evacuated tube Collector (ETC) temp increased to 50 deg C.	947	—	115
Performance evaluation of the senergy polycarbonate and asphalt carbon nanotube solar water heating collectors for building integration	Pugsley et al. (2017)	Senergy polycarbonate CNT (PCNT) and asphalt CNT(ACNT)	FPC and ETC	800	62%	93
Performance analysis of hybrid nanofluids in flat plate solar collector as an advanced working fluid	Verma et al. (2018)	80% MgO and 20% MWCNTs hybrid nanofluid	FPC	800	71.54%	94

		and base fluid water				
Carbon nanotube nanofluid in enhancing the efficiency of evacuated tube solar collector	Mahbubul et al. (2018)	0.2% SWCNT nanoparticle with base fluid water.	ETC	900	66.7%	105
Performance assessment of linear Fresnel solar reflector using MWCNTs_DW nanofluids	Ghodbane et al. (2019)	0.3% MWCNTs nanofluid mixed with distilled water	Linear Fresnel solar reflector	750	33.8%	103
Thermal-hydraulic and thermodynamic performances of liquid metal based nanofluid in parabolic trough solar receiver tube	Peng et al. (2019)	Ga-CNT nanofluids	Parabolic trough collector (PTCs)	1000	45.2%	102
Energy and exergy analysis of a thermosiphon and forced-circulation flat-plate solar collector using MWCNT/water nanofluid	Eltaweel et al. (2019)	Thermosiphn Nanofluid MWCNT/water	FPC size reduced 34% by using 0.1 wt% MWCNTs	From 520 to 914	—	106
		0.01wt%			51.29%	
		0.05 wt%			56.41%	
		0.1 wt%			70.67%	
Comparative investigation of efficiency sensitivity in a flat plate solar collector according to nanofluids	Tong et al. (2020)	0.005 vol% concentration 20 nm MWCNT	FPC	1500	87%	98
The stability, optical properties and solar-thermal conversion performance of SiC-MWCNTs hybrid nanofluids for the direct absorption solar collector (DASC) application	Li et al. (2020)	Ethylene glycol based SiC-MWCNTs nanofluid with concentration of 1 wt% hybrid nanofluid	Direct Absorption Solar Collector (DASC)	— 〃	97.3%	109
An experimental and numerical investigation on a paraffin wax/graphene oxide/carbon nanotubes composite material for solar thermal storage applications	Chen et al. (2020)	Paraffin wax/graphene aerogel composed of graphene oxide and CNT	FPC	1000	73%	96

Augmenting the potable water produced from single slope solar still using CNT-doped paraffin wax as energy storage: experimental approach	Chamkha et al. (2020)	CNT-doped paraffin wax	Phase Change Material (PCM) material thermal energy storage	1010	58.7%	116
Thermal Efficiency, Heat Transfer, and Friction Factor Analyses of MWCNT + Fe3O4_Water Hybrid Nanofluids in a Solar Flat Plate Collector under Thermosyphon Condition	Saleh et al. (2021)	0.3% volume concentration of MWCNTs+Fe_3O_4 based fluid	FPC	785	63.85%	92
A comparison between flat-plate and evacuated tube solar collectors in terms of energy and exergy analysis by using nanofluid	Eltaweel et al. (2021)	0.05% MWCNTs/water fluid	ETC	—	55%	89
Improving environmental performance of a direct absorption parabolic trough collector by using hybrid nanofluids	Mashhadian et al. (2021)	Hybrid nanofluid 0.04 wt% AlO_3/MWCNTs Base fluid water	Direct absorption PTC	856	64.9%	112
The performance response of a heat pipe evacuated tube solar collector using MgO/MWCNT hybrid nanofluid as a working fluid	Henein et al. (2022)	MgO/MWCNT And base fluid is water.	ETC 15 tubes Mass flow rate 1-3L/min	—	55.84%	111
